# A Critical YAP in Malignancy of HCC Is Regulated by Evodiamine

**DOI:** 10.3390/ijms23031855

**Published:** 2022-02-06

**Authors:** Un-Jung Yun, Su-Jin Bae, Yu-Rim Song, Young-Woo Kim

**Affiliations:** School of Korean Medicine, Dongguk University, Gyeongju 38066, Korea; yun2456@dongguk.ac.kr (U.-J.Y.); realsujin@naver.com (S.-J.B.); khf4856@naver.com (Y.-R.S.)

**Keywords:** YAP, AMPK, HCC, evodiamine, liver cancer

## Abstract

Liver cancer has relatively few early symptoms and is usually diagnosed in the advanced stage. Sorafenib is the only first-line anticancer drug approved by the Food and Drug Administration (FDA) for advanced HCC; however, its use is limited due to resistance. Therefore, the development of new drugs is essential to achieving customized treatment. Many studies have suggested that Yes-associated protein (YAP)/transcriptional co-activator with PDZ-binding motif (TAZ) is associated with metastasis and cancer formation and progression in various cancers. In the present study, YAP was overexpressed in various patient-derived hepatocarcinoma (HCC) tissues. In addition, this study examined whether evodiamine (which has anticancer effects) can inhibit YAP and, if so, modulate HCC. Evodiamine significantly reduced both the YAP level and cell growth of HCC in a dose-dependent manner. Biochemical analysis indicated mitochondria dysfunction-mediated apoptosis to be the cause of the reduction in HCC cell growth by evodiamine. YAP was overexpressed in metastatic HCC tissues as well when compared to primary HCC tissues. Migration and invasion analysis showed that evodiamine has anti-metastatic ability on Hep3B and Huh-7 cells and reduces the level of vimentin, an EMT marker. In conclusion, YAP is a critical target in HCC therapy, and evodiamine can be an effective HCC anticancer drug by reducing the YAP level.

## 1. Introduction

Most liver cancer is diagnosed in the advanced stages, decreasing the opportunity for surgical therapies. Hence, the majority of advanced HCC patients have limited therapeutic options. Chemotherapy is one of the most important treatment modalities for advanced HCC [1]. Currently, sorafenib is the only first-line anticancer drug approved by the Food and Drug Administration (FDA) for advanced HCC. The efficacy of sorafenib is brief, and seriously limited because of the development of resistance [2]. Therefore, studies of additional drugs for advanced HCC patients are required to improve the survival of patients.

YAP (Yes-associated protein) and TAZ (transcriptional co-activator with PDZ-binding motif) are considered to be effectors of the Hippo pathway, which is crucial for regulating organ size [3] and cell plasticity in animal development and regeneration [4]. The Hippo pathway inhibits YAP/TAZ through phosphorylation [5]. YAP and TAZ, which are closely related transcriptional regulators, have attracted increasing attention in cancer. YAP/TAZ are hyperactivated in human cancers, and the prolongation of YAP/TAZ activation triggers cancer development [4]. In addition, there is abundant evidence that YAP/TAZ promotes cancer formation, progression, and metastasis. Metastasis occurs when primary cancer cells spread to distinct organs, and is a critical obstacle to cancer therapy. Metastasis manifests as migration, invasion, anchorage-independent growth, epithelial–mesenchymal transition (EMT), and stemness [6]. The functions of YAP/TAZ are critical for the various factors of metastasis. Thus, the activation and critical roles of YAP/TAZ in cancers highlight YAP/TAZ as prime therapeutic targets for anticancer drugs [7].

Evodiamine is a quinazolinocarboline alkaloid and one of the components isolated from the dried, unripe fruit of *Evodia rutaecarpa* (Juss.) Benth. [8]. It affects immunoregulation, anti-angiogenesis, anti-inflammation, inhibitory adipocyte differentiation, and adipogenesis [9]. In addition, studies have shown that evodiamine has antitumor activity in various human cancers [9]. On the other hand, the information on evodiamine is insufficient. This study examined the effects and mechanisms of the antitumor/metastatic activity of evodiamine in HCC in order to assess its potential beneficial use [10].

## 2. Results

### 2.1. YAP Was Upregulated in Liver Tissues

**Figure 1:**
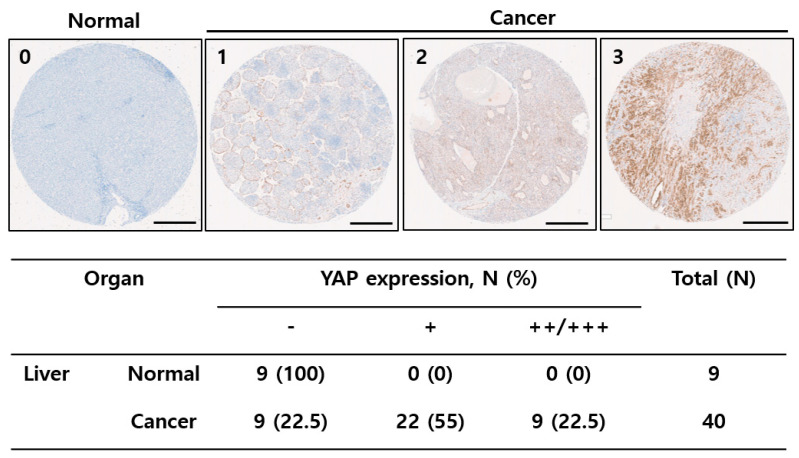
YAP expression in liver tissues and its counterparts. Cancer patient-derived microarrays containing HCC and normal tissues were examined for YAP expression. Scale bar = 500 μm. YAP expression was scored according to the intensity and proportion of positive cells, and representative immunohistochemical images of YAP are shown; none (-), weak (+), moderate (++), or strong (+++). The staining results show the summarized expression of YAP in normal (*n* = 9) and tumor tissues (*n* = 40).

The current interest in YAP has been fueled by the recognition that YAP is widely activated in human tumors and thus an appealing therapeutic target in cancer [7,11]. This study assessed whether YAP could be a drug target for liver cancer as well as the effectiveness of potential YAP inhibitors. First, the YAP levels in tissue microarrays (TMAs) prepared from liver cancer patients were evaluated. Immunohistochemistry analysis showed that the YAP level was higher in liver cancer tissues than in paired normal tissues (Figure 1). Approximately 77.5% of liver cancer tissues were YAP positive, whereas YAP expression in the normal tissues was minimal (Figure 1). These data suggested that YAP could be a drug target in HCC.

### 2.2. YAP Inhibition Induced Apoptosis in HCC Cells

**Figure 2:**
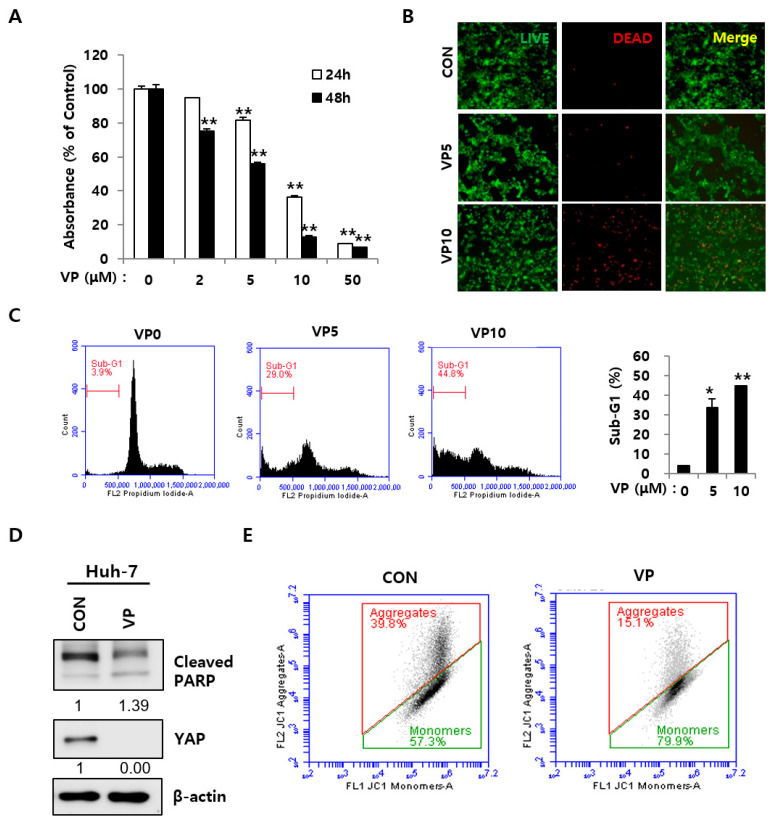
Effects of verteporfin on cell growth and apoptosis of HCC. (**A**) Huh-7 cells were treated with the indicated verteporfin (VP) concentration. Cell growth was measured by a MTT assay at 24 h and 48 h, with error bars representing S.D. (versus control, ** *p* < 0.01). (**B**) Huh-7 cells were treated with the indicated concentration of VP for 48 h, followed by live/dead analysis with calcein-AM (live) and PI (dead). Images were taken by fluorescence microscopy; scale bar = 200 μM. (**C**) Huh-7 cells were treated with the indicated concentration of VP for 48 h followed by cell cycle analysis by flow cytometry. Sub-G1 is summarized in the histogram, with error bars representing S.D. (versus control, * *p* < 0.05, ** *p* < 0.01). (**D**) Huh-7 cells were treated with 20 μM VP for 48 h, followed by immunoblotting analysis with indicated antibodies. (**E**) Huh-7 cells were treated with 10 μM VP for 48 h and stained with JC-1, followed by cell cycle analysis by flow cytometry. The numbers under the western blot indicate the intensity of the band. CON, control; VP, verteporfin.

This study examined whether a YAP inhibitor might be an effective drug target for HCC therapy. First, Huh-7 cells were treated with a YAP-specific inhibitor, verteporfin, and its effects on HCC cells were examined. Verteporfin significantly impaired the cell proliferation of Huh-7 cells (Figure 2A). Next, this study tested whether verteporfin induced cell death in HCC cells. A live and dead cell staining assay was used to stain live (Green) and dead (Red) cells. Verteporfin markedly increased red-stained Huh-7 cells in live/dead staining compared to the control, and cell cycle analysis of Hep3B and Huh-7 revealed the sub-G1 fraction (Figure 2B,C and Supplementary Figure S1A). The accumulation of the sub-G1 population is related to the presence of apoptosis with a reduced DNA content [12]. Verteporfin decreased YAP levels and increased PARP cleavage, which is considered a marker in apoptosis (Figure 2D) [13]. JC-1 staining showed that verteporfin induced mitochondrial dysfunction-mediated apoptotic cell death, as assessed by flow cytometry. The mitochondrial membrane potential (ΔΨm) indicates cell death and functional status, and cytometry analysis with fluorescent probes is used widely to study the mitochondrial behavior [14]. Mitochondrial dysfunction is considered an irreversible step toward apoptosis. JC-1 was used to investigate the mitochondrial membrane potential in Huh-7 cells. JC-1 is a unique fluorescent probe forming red-aggregates under a high ΔΨm, whereas the monomeric green form was increased in cells with ΔΨm [15]. Verteporfin increased green monomers in Huh-7 cells significantly (Figure 2E). This study then investigated whether siRNA-mediated YAP knockdown induced apoptotic cell death in order to confirm the effects of YAP inhibition. Two different YAP-targeting siRNAs showed cell death morphology under a microscope as well as an increase in the monomeric green form in JC-1 staining (Supplementary Figure S1B,C). These results suggest that YAP inhibition could hinder HCC growth through mitochondrial membrane dysfunction-mediated apoptosis.

### 2.3. Evodiamine Decreased YAP Levels and Inhibited the Cell Growth of HCC Cells

**Figure 3:**
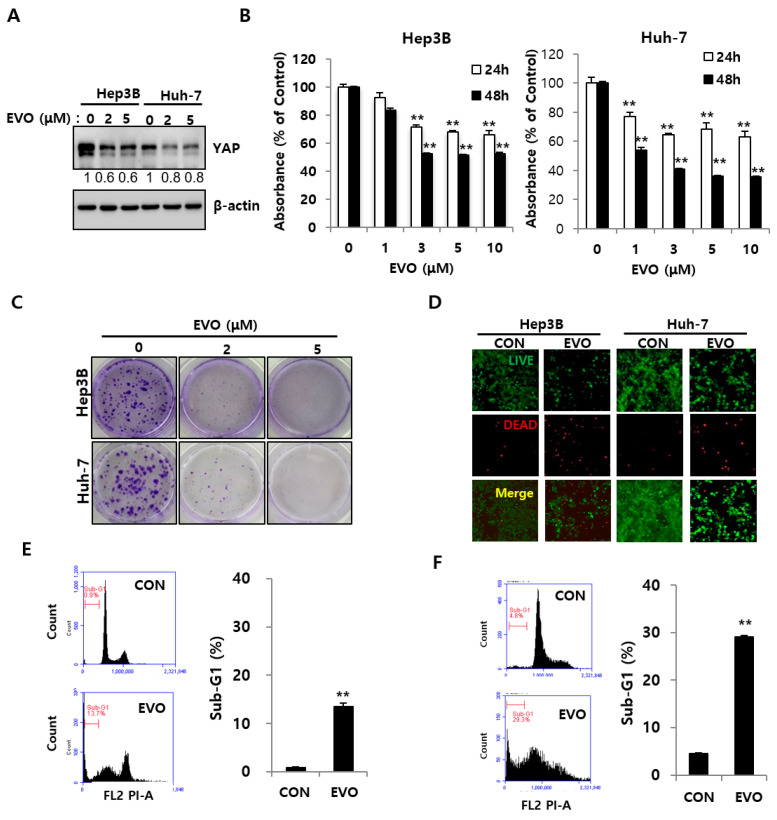
YAP regulation and cell death by evodiamine. (**A**) Hep3B and Huh-7 cells were treated with the indicated concentration of evodiamine for 48 h, followed by immunoblotting analysis with YAP and β-actin antibodies. The loading control was β-actin. (**B**) Hep3B and Huh-7 cells were treated with the indicated concentration of evodiamine (EVO). Cell growth was measured using a MTT assay at 24 h and 48 h, with error bars representing S.D. (versus control, ** *p* < 0.01) (**C**) Hep3B and Huh-7 cells were treated with the indicated concentration of evodiamine (EVO) for 48 h, which was then changed with fresh media without EVO and incubated for ten days. (**D**) Hep3B and Huh-7 cells were treated with 5 μM EVO for 48 h, followed by live/dead analysis with calcein-AM (live) and PI (dead). Images were taken by fluorescence microscopy. Scale bar = 200 μM. (**E**,**F**) Hep3B (**E**) and Huh-7 (**F**) cells were treated with 5 μM EVO for 48 h, followed by cell cycle analysis by flow cytometry. The Sub-G1 is summarized in the histogram, with error bars representing S.D. (versus Control, ** *p* < 0.01). The numbers under the western blot indicate the intensity of the band. CON, control; EVO, evodiamine.

YAP is frequently overexpressed in many human cancers, and can expand the liver size and drive cancer development in mouse models [16,17]. Previous studies have shown that YAP is a critical mediator of apoptosis [18,19]. The change in YAP levels caused by evodiamine was examined in Hep3B and Huh-7 cells. Evodiamine reduced the YAP level in HCC cells significantly (Figure 3A). The effects of evodiamine on the growth of Hep3B and Huh7 were explored as well. Cell proliferation was measured using an MTT assay. Evodiamine (1–10 μM) reduced cell growth of Hep3B cells significantly compared to the control; the IC 50 was about 1.5 μM in the Huh-7 at 48 h (Figure 3B). It reduced cell growth of Huh-7 cells from 77.2% to 63.1% for 24 h and from 53.8% to 35.4% for 48 h compared to the control (Figure 3B). The cell proliferation assay revealed evodiamine to be more effective on Huh-7 than on Hep3B. A colony formation assay was used to assess the effect of evodiamine on long-term survival. A colony formation assay is an in vitro cell survival assay that assesses the ability of a single cell to grow into a colony [20]. Evodiamine reduced the long-term survival of Hep3B and Huh-7 cells (Figure 3C). Moreover, evodiamine (20 and 50 μM) reduced cell proliferation and long-term survival in highly aggressive HepG2 cells according to both the MTT assay and the colony formation assay (Supplementary Figure S2A,B). A live/dead cell staining assay was performed using 5 μM evodiamine. Evodiamine markedly increased red cells as a result of cell death in Hep3B and Huh-7 cells compared to Hep3B and Huh-7 cells in the control (Figure 3D) in the live/dead assay. Cell cycle analysis showed that evodiamine increased the sub-G1 apoptotic proportion in the Hep3B and Huh-7 cells (Figure 3E,F).

### 2.4. Evodiamine Induced Apoptosis via Mitochondrial Dysfunction

**Figure 4:**
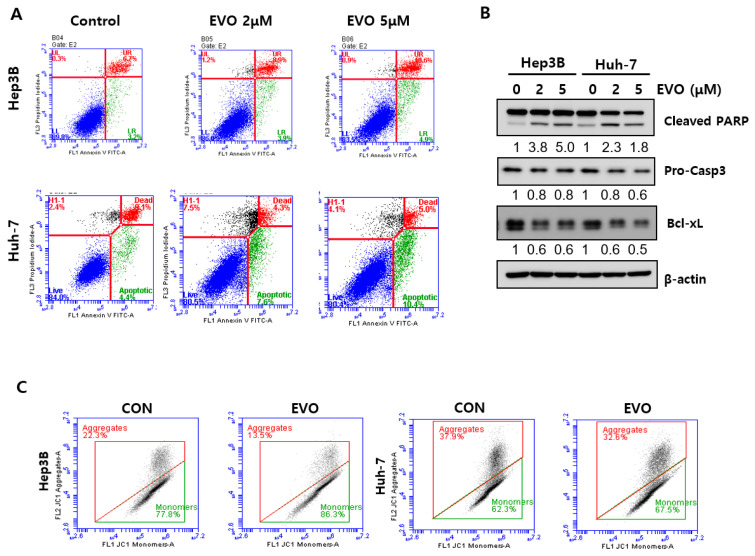
Apoptosis induction by evodiamine. (**A**,**B**) Hep3B and Huh-7 cells were treated with the indicated concentration of evodiamine for 48 h, stained with Annexin V/PI, and analyzed by flow cytometry (**A**). This was followed by immunoblotting analysis with the indicated antibodies. The loading control was β-actin (**B**). (**C**) Huh-7 and Hep3B cells were treated with the indicated concentration of evodiamine for 48 h and stained with JC-1, analyzed by flow cytometry. The numbers under the western blot indicate the relative intensity of the band. CON, control; EVO, evodiamine.

Annexin V/PI staining, which is used for determining apoptosis, was performed to evaluate apoptosis as a possible mechanism for cell death induction by evodiamine [21]. Evodiamine increased the number of early apoptotic (annexin V+/PI-) and late apoptotic dead cells (annexin V+/PI+) in a dose-dependent manner (Figure 4A). Consistent with these results, evodiamine also cleaved PARP and caspase 3 and decreased the anti-apoptotic protein Bcl-xL in HCC cells in a dose-dependent manner (Figure 4B and Supplementary Figure S2B) [22]. Evodiamine increased the proportion of the monomeric green form in Hep3B and Huh-7 cells (Figure 4C). Overall, evodiamine induces apoptosis by reducing the mitochondrial membrane potential, and YAP mediates the effects of evodiamine in the apoptosis of HCC cells.

### 2.5. Evodiamine Modulated EMT Ability by Regulating YAP in HCC

**Figure 5:**
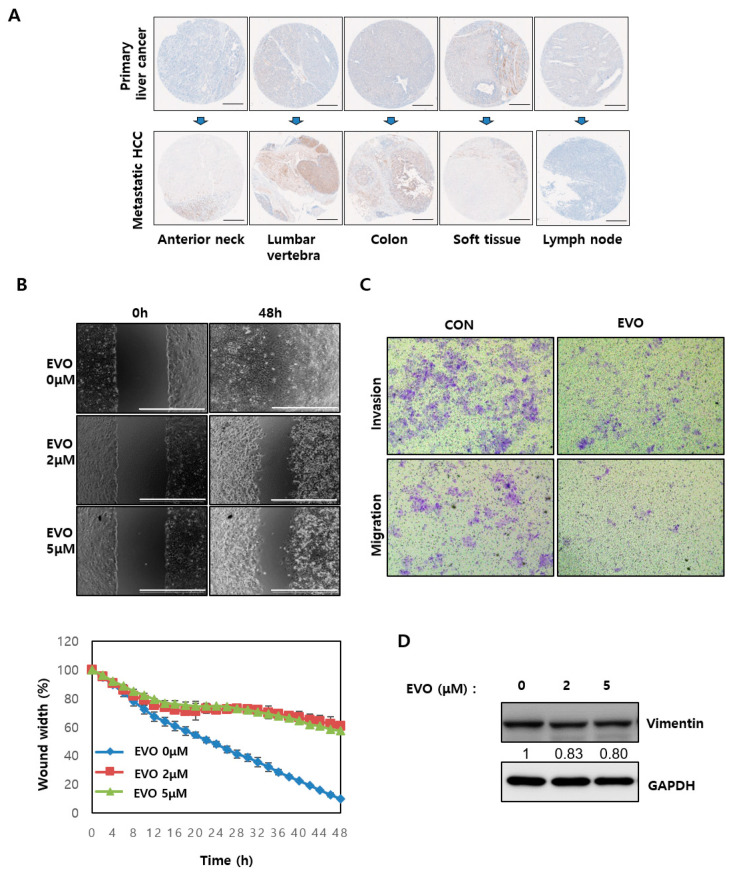
Effects of evodiamine on the metastatic ability of HCC. (**A**) Cancer patient-derived microarrays containing primary HCC and its metastatic HCC tissues were examined for YAP expression. Staining results were graded according to the intensity and proportion of positive cells as described in ‘Materials and Methods’. Scale bar = 500 μm. (**B**) Hep3B cells were scrapped and incubated with 0, 2, and 5 μM EVO for 48 h. The scrapped scratches were imaged using an optical microscope. The data are expressed as the percentage of wound width. Scale bar = 1000 μm. (**C**) Hep3B cells were treated with 5 μM EVO for 24 h and plated on transwell coated with matrigel (invasion) or without matrigel (migration). (**D**) Hep3B cells were treated with the indicated concentration of evodiamine for 48 h, followed by immunoblotting analysis with vimentin and GAPDH antibodies. The numbers under the western blot indicate the intensity of the band. CON, control; EVO, evodiamine.

Recurrence and metastasis are major factors affecting the poor prognosis of HCC [22]. Recent reports have shown that YAP is associated with metastasis in cancer by promoting EMT and increasing the cell mobility [23,24,25]. Consistent with these reports, the YAP levels were higher in various metastatic HCC tissues than primary HCC in the microtissue array analysis (Figure 5A). The effects of evodiamine on the metastatic ability of Hep3B and Huh-7 HCC cells were tested. In the wound-healing assay, a confluent monolayer of the cell was scratched and treated with evodiamine and optical microscopy images were taken. In the control, 10.07% closure of the scratch gap was noted; however, evodiamine only closed 57.5% of the scratch gap in Hep3B cells (Figure 5B). The results of wound-healing assay in Huh-7 cells were lower than that of Hep3B cells (Supplementary Figure S3A). The wound-healing assay showed that evodiamine impaired wound closure in HCC cells, suggesting that the migratory ability of HCC cells was decreased by evodiamine. Similarly, evodiamine inhibited the migration and invasion of Hep3B and Huh-7 cells in the transwell (Figure 5C and Supplementary Figure S3B). Evodiamine decreased the levels of vimentin, an EMT (Epithelial–mesenchymal transition) marker, in HCC cells (Figure 5D and Supplementary Figure S3C). Overall, these results show that YAP is a critical target in metastatic HCC. Moreover, evodiamine can be a potential therapeutic drug for metastatic HCC.

## 3. Discussion

HCC is one of the most common cancers with high mortality [26]. Only 15% of patients with HCC can undergo a surgical resection, and liver transplantation is considered a potentially curative approach [26]. The majority of patients proceed to advanced disease. In advanced disease, conventional sorafenib is the only treatment; however, its efficacy is limited by the development of resistance [2]. In addition, the metastasis of HCC is a critical cause of poor prognosis. Thus, exploring additional drugs available for advanced HCC patients is essential. This study suggests that evodiamine can potentially be a novel anticancer drug through inhibition of metastatic behavior in HCC via YAP.

Hippo signaling, a prime regulator of cancer, regulates its nuclear/cytoplasmic distribution by phosphorylating YAP/TAZ, and its nucleo-cytoplasmic shuttling induces expression of cell-proliferative and anti-apoptotic genes by regulating various transcription factors [27]. YAP is frequently activated during hepatocarcinogenesis in lung, colorectal, breast, pancreatic, melanoma, and glioma cancers [28,29]. This study showed that while YAP was overexpressed in HCC, YAP expression was minimal in normal tissues, although the numbers of normal cases are relatively small (Figure 1). YAP/TAZ integrate various oncogenic signaling pathways such as EGFR, TGFβ, Wnt, PI3K, GPCR, and KRAS, and are instrumental for tumor initiation, progression, and chemoresistance [3]. Furthermore, YAP/TAZ contribute to cancer-stem cells (CSC), preserve the stemness properties, and generate new CSCs [7]. On the other hand, recent studies suggested that YAP has tumor suppressor roles in suppressing WNT signaling and triggering DNA damage-induced apoptosis [27]. In particular, YAP/TAZ may contribute differentially to tumorigenesis depending on the cellular context. Nevertheless, various studies on the activation of YAP in carcinomas, the crucial role of YAP activation, and the poor prognosis indicated by YAP suggest YAP as a prime target for screening and design of anticancer drugs [7].

Evodiamine has potential anticancer activity in human cancer cells. Reports have shown its effects on various cancer cell lines. With Zhao et al. having shown that evodiamine inhibits proliferation and promotes apoptosis of HCC, we focused on the role of YAP in the human tissue and related signaling pathways [30]. First, this study examined whether evodiamine regulates YAP levels in Hep3B and Huh-7. Evodiamine reduced the YAP levels significantly (Figure 1 and Figure 2). Judging from PARP cleavage, JC-1staining, and live/dead staining, YAP inhibition caused mitochondrial dysfunction-mediated apoptosis (Figure 2). Chemotherapeutic drugs inducing apoptosis depend mainly on the changes in the mitochondrial status [31]. The evodiamine treatment accelerated mitochondrial dysfunction-mediated apoptosis by inducing PARP and Caspase 3 cleavage in HCC cells (Figure 3). Li et al. reported that YAP could regulate anti-apoptotic Bcl-xL [31]. Bcl-xL is a member of the anti-apoptotic Bcl-2 family, which is localized in the mitochondria [32]. Bcl-xL is an oncogene overexpressed in many cancers and the key regulator of the mitochondrial pathway [32]. Therefore, YAP/Bcl-xL is a potential target in therapeutic cancer strategy. Evodiamine treatment decreased Bcl-xL, with a concomitant decrease in YAP levels (Figure 2A and Figure 4B).

Metastasis is a major obstacle to successful cancer treatment and is responsible for cancer-related death. YAP/TAZ activation promotes several steps in metastasis, including epithelial-to-mesenchymal cell transition (EMT), invasion, migration, intravasation, anoikis resistance, and immune evasion. Moreover, it has been implicated in the metastasis of numerous cancer types [24]. In support of this notion, this study examined YAP levels in primary HCC cancer tissues and metastatic HCC cancer tissues. The YAP level was increased in the metastatic HCC cancer tissues compared to the primary HCC tissues (Figure 4A). Clinical analysis showed that this was consistent with the leading tendency of recent evidence showing that YAP is closely associated with metastasis. These results led the authors to investigate whether the YAP regulator evodiamine regulates metastatic ability. As expected, evodiamine decreased the migration and invasion of HCC cells (Figure 4 and Supplementary Figure S3). EMT is a highly conserved developmental program that converts polarized, immotile epithelial cells to cells with motile mesenchymal properties [33,34]. It is a crucial driver in metastasis [35]. The mesenchymal features promote the motility and invasiveness of malignant cells [33]. EMT is characterized by the enhanced expression of the mesenchymal marker N-cadherin and by vimentin progressing to a metastatic phenotype. Evodiamine reduced the expression of vimentin (Figure 5D and Supplementary Figure S3D). Evodiamine treatment inhibited EMT, which has a critical role in metastasis.

Multiple studies have shown that YAP is associated with cancer resistance to various treatments including chemotherapy, radiation therapy, and targeted therapy [28,36]. Kim et al. reported that YAP is involved in suppressing the antitumor immune response through YAP-induced PD-L1 upregulation [37]. Either knockdown or pharmacological inhibition of YAP sensitizes esophageal cancer cells towards 5-FU and docetaxel [38] and potentiates the DNA damage response and apoptosis after γ-irradiation [39]. YAP induced BRAFi resistance through overexpression of the BCL-xL gene, and verteporfin, a YAP inhibitor, sensitized the resistant melanoma cells to BRAFi [40]. Therefore, YAP inhibition can be an important target in overcoming cancer resistance. Furthermore, inhibitors of YAP/TAZ transcriptional targets such as CTGF, AXL, and Bcl-xL, are undergoing clinical trials in combination with standard therapies [38]. As mentioned previously, evodiamine inhibited HCC cancer via YAP/Bcl-xL. Based on these results, evodiamine can sufficiently overcome anticancer drug resistance. On the other hand, the efficacy of evodiamine against cancer resistance was not explored. A future study will explore the effectiveness and mechanism of evodiamine on anticancer drug resistance.

Among YAP/TAZ inhibitors only verteporfin acts directly on YAP/TAZ, despite more than fifty drugs having been shown to inhibit YAP/TAZ activity [3]. While direct YAP/TAZ inhibitors are currently under development, they are not clinically viable YAP inhibitors [36]. As YAP/TAZ contributes to the acquisition of cancer traits, the strategy of targeting them is essential for managing HCC. Therefore, in addition to developing new drugs against YAP/TAZ, it is essential to determine whether known drugs can be recycled as YAP/TAZ-inhibitors.

## 4. Materials and Methods

### 4.1. Chemicals and Reagents

Anti-PARP, caspase 3, Bcl-xL, LC3B, and β-actin antibodies were purchased from Cell Signaling (Beverly, MA, USA). Anti-YAP antibodies were supplied by Santa Cruz Biotechnology (Santa Cruz, CA, USA). Horseradish peroxidase (HRP)-tagged goat anti-mouse and goat anti-rabbit IgGs were obtained from (Enzo Life Sciences, Farmingdale, NY, USA). 3-(4,5-dimethylthiazol-2-yl)-2,5-diphenyl-tetrazolium bromide (MTT) and dimethyl sulfoxide (DMSO) were acquired from Sigma-Aldrich (St. Louis, MO, USA).

### 4.2. Cell Lines and Cell Culture

Hep3B and Huh-7 human HCC cells were procured from the Korean Cell Line Bank (KCLB, Seoul, Korea). Hep3B and Huh-7 were cultured in DMEM high glucose or RPMI 1640 medium with 10% FBS and 50 units/mL penicillin and 50 μg/mL streptomycin 37 °C in a humidified atmosphere containing 5% CO_2_, respectively.

### 4.3. Cell Viability Assay

For the cell proliferation assay, the cells were plated in a 96-well culture plate for 24 h before treatment (approximately 70% confluence). Cell growth was determined using an MTT [3-(4,5-dimethylthiazol-2-yl)-2,5-diphenyltetrazolium bromide] assay for 2 h at 37 °C in a humidified atmosphere containing 5% CO_2_. Following incubation the medium was removed and 100 μL of DMSO was added to each well in order to dissolve the formazan crystals. The absorbance was measured at 570 nm using an ELISA microplate reader (Tecan, Research Triangle Park, NC, USA). Cell viability was measured by exposing the cells to the indicated concentrations of evodiamine in DMEM or RPMI 1640 medium. After incubation, the cells were stained with 0.5 µg/mL green-fluorescent calcein-AM and red-fluorescent propidium iodide (PI).

### 4.4. Flow Cytometry

The cells were treated and stained with annexin V/propidium iodine (PI, BD Bioscience, San Jose, CA, USA) according to the manufacturer’s protocol. For cell cycle analysis, the cells were harvested, fixed with 70% ethanol, and stained with a PI solution (20 μg/mL PI, 0.1% sodium citrate, 50 μg/mL RNase A, 0.03% NP-40, PBS). The stained cells were analyzed using an Accuri C6 flow cytometer (Accuri Cytometers Inc., Ann Arbor, MI, USA) [10].

### 4.5. Immunoblotting Analysis

The cell extracts were prepared by incubating the cells with a lysis buffer (Thermo, Rockford, IL, USA) at 4 °C. The supernatant was collected by centrifugation at 15,000 rpm for 30 min at 4 °C. The immune complexes were visualized using the enhanced chemiluminescence method with ECL reagent (Advensta, Menlo Park, CA, USA) and a chemi-doc image analyzer (Vilber Lourmat, Collégien, France) [41].

### 4.6. Mitochondrial Membrane Potential (ΔΨm) Analysis

The mitochondrial transmembrane potential (ΔΨm) was measured using JC-1, a fluorescent carbocyanine dye, according to the protocol described previously [42]. The cells were plated in a six-well plate and treated with different drug concentrations. After treatment, the cells were incubated with 20 μM JC-1 in media for 30 min, then subjected to flow cytometry analysis to measure the JC-1 aggregate (red) to monomer (green) ratio.

### 4.7. Clonogenic Assay

Cells were plated at 5 × 10^2^ per six-well plate and treated with evodiamine for 48 h. The cells were incubated for another ten days in the drug-free medium. The cells were fixed with 4% formalin and stained with a 1% crystal violet solution, then air-dried.

### 4.8. Immunohistochemical Staining for the Cancer Tissue Microarray

Tissue arrays were obtained from Superbiochips Laboratories (Seoul, Korea). The slide included specimens of HCC, metastatic HCC, and liver normal tissues obtained by biopsy or surgical resection from patients. Immunohistochemical staining was performed using a BenchMark XT automated slide stainer (Ventana Medical Systems, Inc., Tucson, AZ, USA) and OptiView DAB IHC detection Kit (Ventana). The slides were counterstained with hematoxylin (760-2021; Ventana Medical Systems, Inc., Tucson, AZ, USA) and Bluing reagent (760-2037; Ventana Medical Systems, Inc., Tucson, AZ, USA).

### 4.9. Scratch Wound Healing Assay

The cells were plated on six-well plates when they had reached 90% confluence. The monolayer was scratched, and the cells were incubated without or with various concentrations of evodiamine for 24 h and 48 h. The cells were observed under Automated Microscope (Bio-Tek lionheart, Winooski, VT, USA).

### 4.10. Cell Invasion Assay

The cells were plated onto a six-well dish and treated with the indicated evodiamine concentration. The cells treated with evodiamine were mixed with serum-free media and added to the upper chamber coated with matrigel (invasion) or without matrigel (migration). Complete medium containing 10% FBS was added to the lower chamber. After incubation for 18 h, the cells in the upper chamber were removed, and cells traversed through the membrane were fixed with 4% formaldehyde and stained with 1% crystal violet.

### 4.11. Statistical Analysis

The experimental results are presented as the mean ± standard deviation (S.D.) of experiments repeated at least three times. For each significant treatment effect, ANOVA or T-test was used to compare the multiple group means. The criterion for statistical significance was set as *p* < 0.05 or *p* < 0.01.

## 5. Conclusions

In conclusion, evodiamine decreased YAP/Bcl-xL levels in HCC cells, inducing mitochondria dysfunction-mediated apoptosis. Furthermore, evodiamine blocked the mesenchymal features promoting the motility and invasiveness of malignant HCC cells. Overall, this study suggests that evodiamine might be a candidate as a new YAP inhibitor for HCC therapeutic strategies. Nevertheless, more meticulous research on the mechanisms acting on YAP as well as *in vivo* experiments using nude mice will be needed.

## Data Availability

The data presented in this study are available on request from the corresponding author.

## Supplementary Materials

The following supporting information can be downloaded at: https://www.mdpi.com/article/10.3390/ijms23031855/s1.
