# Supplementary material for: A Critical YAP in Malignancy of HCC Is Regulated by Evodiamine
Source: Int J Mol Sci. 2022 Feb 6;23(3):1855. doi: 10.3390/ijms23031855 (PMC8837083; doi:10.3390/ijms23031855)
Supplement: Supplementary file 1 [file ijms-23-01855-s001.zip › ijms-1557290-supplementary.pdf]

Supplementary Information

Supplementary Material and Methods

Reagents and antibodies

Anti-PARP, caspase 3, vimentin, and β-actin antibodies were purchased from purchased from Cell Signaling (Beverly, MA, USA). Anti-YAP antibodies were purchased from purchased from Santa Cruz Biotechnology (Sannta Cruz, CA, USA). Horseradish peroxidase (HRP)-tagged goat anti-mouse and goat anti-rabbit IgGs were obtained from (Enzo Life Sciences, Farmingdale, USA). 3-(4,5-dimethylthiazol-2-yl)-2,5-diphenyl-tetrazolium bromide (MTT) and dimethyl sulfoxide (DMSO) and evodiamine were purchased from Sigma-Aldrich (St. Louis, MO, USA).

Cell culture

Hep3B, Huh7 and HepG2 human hepatocellular carcinoma cells were purchased from the Korean Cell Line Bank (KCLB, Seoul, Korea). Hep3B and HepG2 were cultured in DMEM high glucose and Huh-7 cells were cultured in RPMI 1640 medium with 10% FBS and 50 units/ml penicillin and 50 μg/ml streptomycin 37 °C in a humidified 5% CO2 atmosphere, respectively.

Cell proliferation assay (MTT)

For the cell proliferation assay, cells were plated in a 96-well culture plate for 24 h before treatment (approximately 70% confluence). Cell growth was determined MTT [3-(4,5-dimethylthiazol-2-yl)-2,5-diphenyltetrazolium bromide] for 2 h at 37 °C in a humidified 5% CO2 atmosphere. Following incubation, the cells were stained with 0.5ug/ml green-fluorescent calcein AM and red-fluorescent propidium iodide (PI)

siRNA transfection

siRNA duplexes into cells were performed using lipofectamine RNAimax (Invitrogen, Carlsbad, CA, USA) reversely as described by the manufacturer. A scrambled siRNA was used as a negative control (NC, SN-1002, Bioneer, Korea). Sequences for REP1 #1 and #2 were 5′-CAGAAGAUCAAAGCUACUU -3′ and 5′-AGAACCGUUUCCCAGACUA-3′, respectively.

Flow cytometry analysis

Cells were treated and stained with annexin V / propidium iodine (BD Bioscience, San Jose, CA) according to manufacturer's protocol. For the cell cycle analysis, cells were harvested, fixed with 70% ethanol, and stained with propidium iodide (PI) solution (20 μg/ml PI, 0.1% sodium citrate, 50 μg/ml RNase A, 0.03% NP-40, PBS) The stained cells were analyzed by Accuri C6 flow cytometer (Accuri Cytometers Inc., Ann Arbor, MI, USA) (1).

Immunoblotting analysis

Cell extracts from treated cells as described were prepared by incubating at 4 °C with Lysis Buffer (Thermo, Rockford, U.S.A.). The immune complexes were visualized using the enhanced chemiluminescence method using ECL reagent (Advensta, Menlo Park, CA, USA) and a chemi-doc image analyzer (Vilber Lourmat, France) (2).

Mitochondrial Membrane Potential (ΔΨm) analysis

Mitochondrial transmembrane potential (ΔΨm) was measured using JC-1, a fluorescent carbocyanine dye by the previously described protocol (3). Cells were plated in a 6-well plate and treated with different concentrations of drug. After treatment, cells were incubated with 20 μM JC-1 in media for 30 min, and then cells were subjected to flow cytometry analysis to measure the ratio of JC-1 aggregate (red) to monomer (green).

Clonogenic assay

Cells were plated at 5×10^2 per 6-well plate and treated with evodiamine for 48 h. The cells were incubated for another 10 days in the drug-free medium. The cells were fixed with 4% formalin and stained with a 1% crystal violet solution, and then air-dried.

Scratch wound healing assay

Cells were plated on 6-well plates at 90% confluence. The monolayer was scratched and then were incubated with or without various concentrations of evodiamine for 24h and 48h. Cells

were observed under Automated Microscope (Bio-tek lionheart, Winooski, VT, USA).

Cell migration and invasion assay

Cells were plated onto 6-well dish and treated with indicated concentration of evodiamine. Then cells treated with evodiamine was mixed with serum-free media and added into the upper chamber coated with/without matrigel. Complete media containing10% FBS were added into the lower chamber. After incubation for 18hr, cells in upper chamber were removed and cells traversed through membrane were fixed with 4% formaldehyde, stained with 1% crystal violet.

Statistical analysis

The experimental results were presented as mean±standard deviation (S.D.) of experiments repeated at least three times. For each significant treatment effect, the ANOVA or T-test was utilized to compare multiple group means. The criterion for statistical significance was set at $P<0.05$ or $P<0.01$.

Supplementary references

1. Yun UJ, Lee IH, Lee JS, Shim J, Kim YN. Ginsenoside Rp1, A Ginsenoside Derivative, Augments Anti-Cancer Effects of Actinomycin D via Downregulation of an AKT-SIRT1 Pathway. Cancers (Basel). 2020;12(3).

2. Yun UJ, Lee JH, Koo KH, Ye SK, Kim SY, Lee CH, et al. Lipid raft modulation by Rp1 reverses multidrug resistance via inactivating MDR-1 and Src inhibition. Biochem Pharmacol. 2013;85(10):1441-53.

3.Li C, Liu H, Yang Y, Xu X, Lv T, Zhang H, et al. N-myristoylation of Antimicrobial Peptide CM4 Enhances Its Anticancer Activity by Interacting With Cell Membrane and Targeting Mitochondria in Breast Cancer Cells. Front Pharmacol. 2018;9:1297.

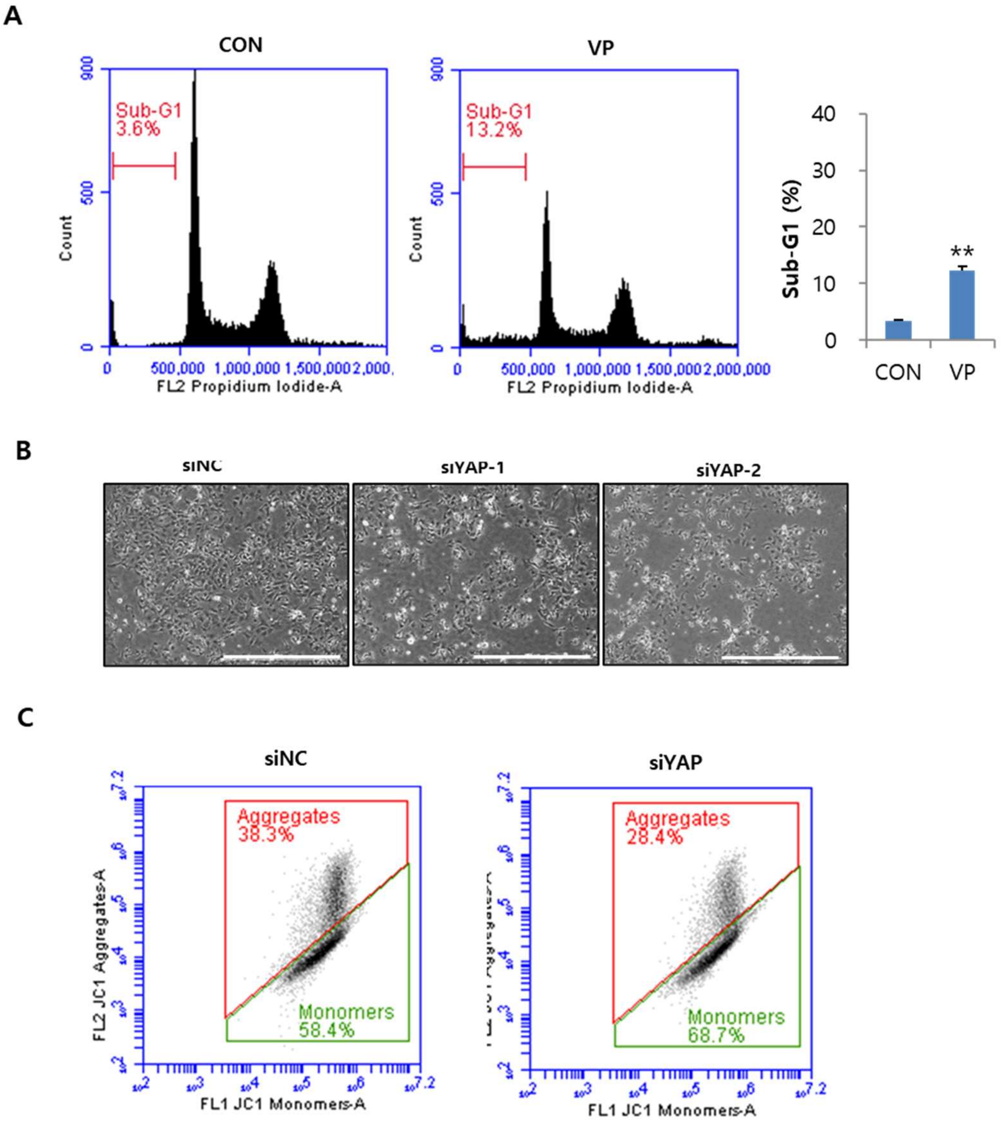

Supplementary Figure S1. Effects of YAP down regulation on HCC cells. (A) Hep3B cells were treated with 20 M VP for 24h, followed cell cycle analysis by flow cytometry. Sub-G1 was summarized in the histogram, with error bars representing S.D. (versus Control, ***P*<0.01). **(B)** Hep3B cells were transfected with either siNC or two different siYAP. Cell images were taken using phase contrast microscopy. Scale bar=1000 M. **(C)** Hep3B cells were transfected with either siNC or siYAP and incubated for 48hr. After JC-1 staining, cells were followed cell cycle analysis by flow cytometry. This is confirmed by repeated experiments at least 3 times. CON, control; VP, verteporfin

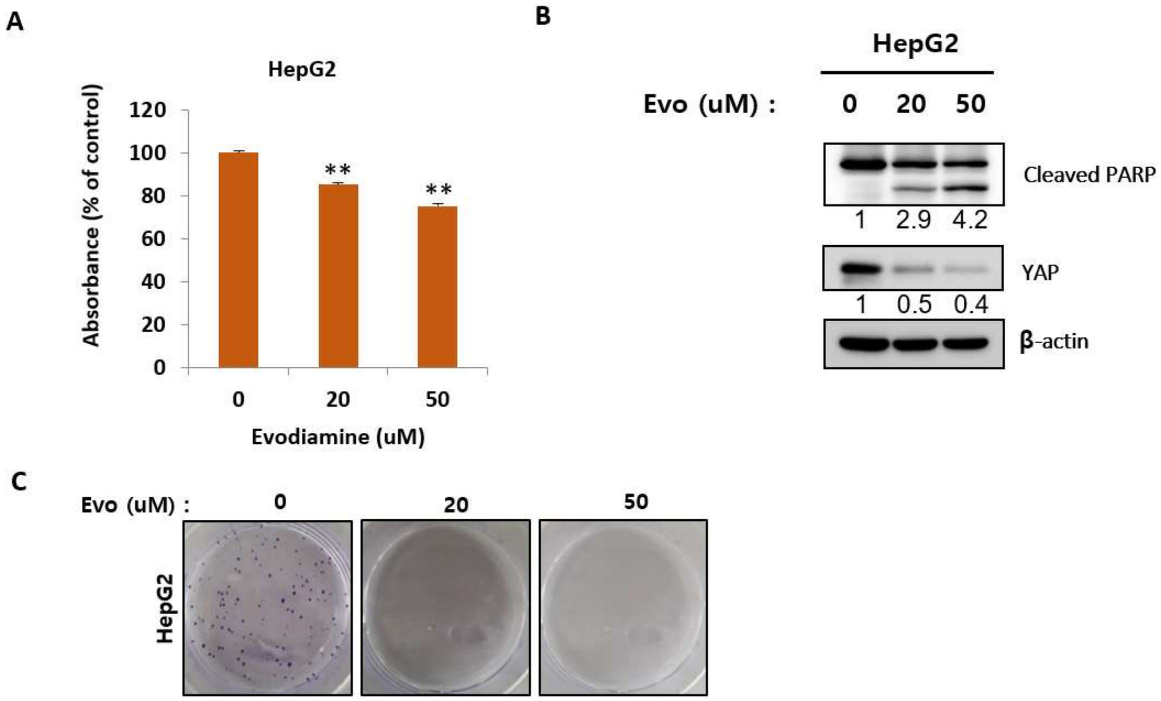

Supplementary Figure S2. YAP regulation and cell death by evodiamine in HepG2 cells. (A) HepG2 cells treated with indicated concentration of evodiamine (EVO). Cell growth was measured by MTT assay at 48h, with error bars representing S.D. (versus control, ***P*<0.01). **(B)** HepG2 cells were treated with indicated concentration of evodiamine for 48h, followed by immunoblotting analysis with YAP, PARP and -actin antibodies. **(C)** HepG2 cells were treated with indicated concentration of evodiamine for 48h, subjected to clonogenic assay. The numbers under the western blot indicate the intensity of the band. CON, control; VP, verteporfin

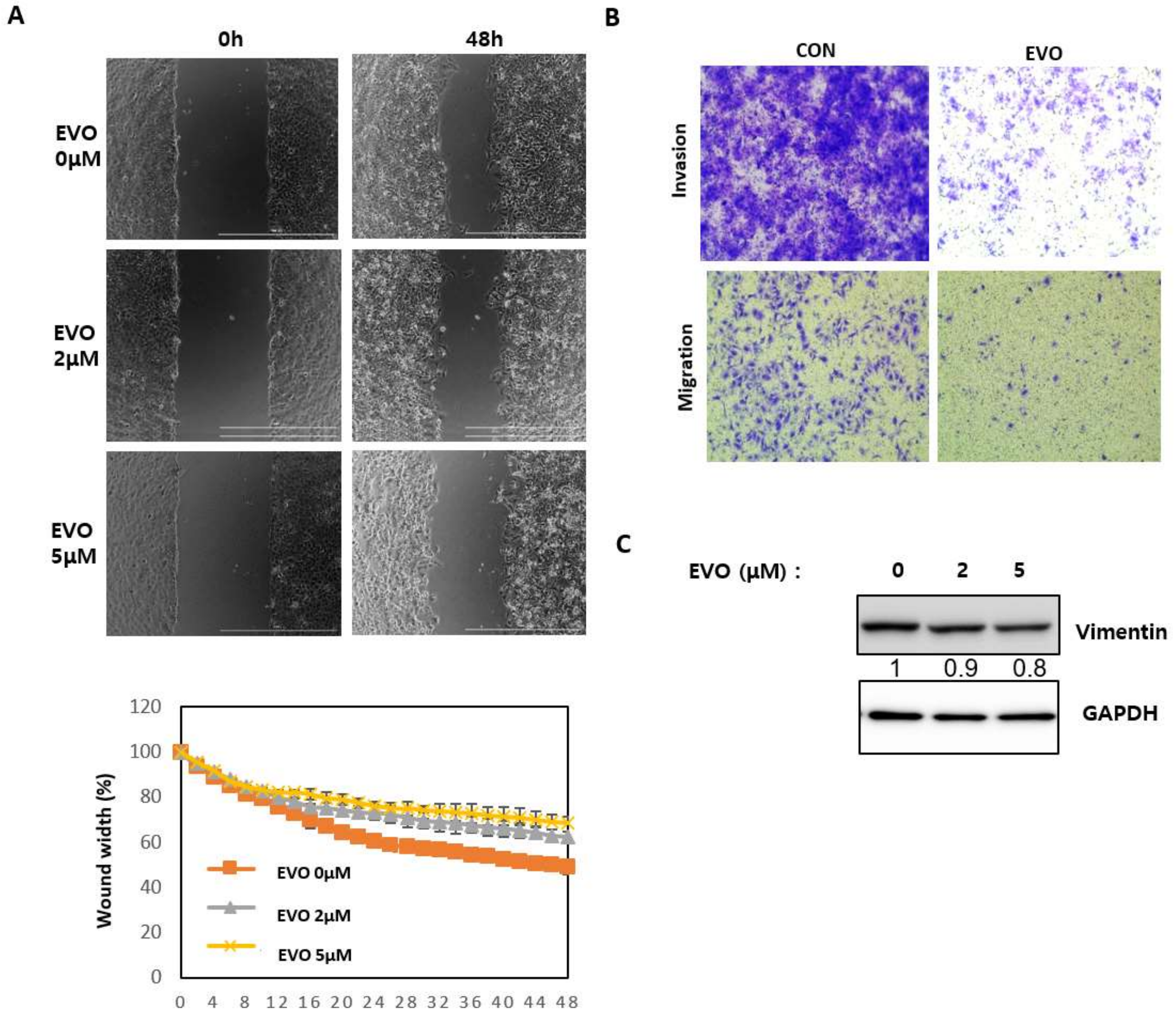

Supplementary Figure S3. Effects of evodiamine on metastatic ability of Huh-7 cells. (A) Huh-7 cells were scrapped and incubated with 0, 2, and 5 μM EVO for 48 h. scrapped scratches were captured using light microscope. Data are expressed in percentage of wound width. Scale bar = 1000 μm. **(B)** Huh-7 cells were treated 5 M EVO for 24 h and plated on transwell coated with/without Matrigel. **(C)** Huh-7 cells were treated with indicated concentration of evodiamine for 48h, followed by immunoblotting analysis with vimentin and GAPDH antibodies. The numbers under the western blot indicate the intensity of the band. CON, control; VP, verteporfin
